# Development of Critical Reflection Competency Scale for Clinical Nurses

**DOI:** 10.3390/ijerph19063483

**Published:** 2022-03-15

**Authors:** Sujin Shin, Eunmin Hong, Jiyoung Do, Mee Sun Lee, Youngsun Jung, Inyoung Lee

**Affiliations:** 1 College of Nursing, Ewha Womans University, Seoul 03760, Korea; ssj1119@ewha.ac.kr (S.S.); thename126@gmail.com (E.H.); djy0221@naver.com (J.D.); ms5723460@naver.com (M.S.L.); 2 Department of Nursing, Asan Medical Center, Seoul 05505, Korea; happy6702@hanmail.net

**Keywords:** critical reasoning, nurses, thinking, education, nursing education research

## Abstract

Critical reflection develops nurses’ critical thinking and clinical reasoning competency. It is necessary to develop a validated scale to measure critical reflection competency considering the clinical situation and nursing context. Therefore, this study analyzed the concept of critical reflection, developed a scale to measure critical reflection competency, and verified its validity and reliability. The concept of critical reflection and components of the scale were confirmed through literature review and results of previous studies on content analysis. A total of 64 preliminary items were derived on a 5-point Likert scale. The adequacy of vocabulary and expression was checked, and a content validity test was conducted. An I-CVI value of 0.88–1.00 was computed. Construct validity was conducted through an exploratory factor analysis, and data collected from 296 clinical nurses were analyzed. Five factors and nineteen items were derived, and the explanatory power was found to be 53.02%. Cronbach’s α of the scale was 0.853. Future studies need to develop a critical reflection education program and utilize this concept as an educational strategy. We propose a study to verify the effect of applying an educational program using the critical reflection competency scale developed in this study.

## 1. Introduction

Critical reflection can be used as an educational strategy that systematically integrates experiences, praxes, and theories in clinical practice [1]. It narrows the gap between theory and practice and improves professional development and nursing practice based on nurses’ experience, because it helps them critically evaluate and change their nursing practice [2]. Through critical reflection, nurses reflect on their experiences and ask the “why” question about the nursing situation, expanding their thinking and understanding the context of the situation in depth. Deep learning that connects theory and practice occurs through critical reflection, which allows nursing practice to be applied and developed in a desirable way [3,4]. Critical reflection helps nurses understand and judge clinical situations based on evidence and enhances their problem-solving ability [4]. It also provides insight into the clinical situation and one’s own nursing performance through metacognition, reduces the risk of errors [5], and improves communication with patients and colleagues [6,7]. In addition, it helps nurses deeply understand and effectively manage the negative emotions and stresses they experience at work [8,9]. In particular, critical reflection can be facilitated by positive feedback and can prove useful in forming positive relationships with colleagues and adjusting to work, because it is based on an open attitude [3,4]. As such, it is important for clinical nurses to develop critical reflection competency because it can promote their individual growth by developing nursing work competency and professionalism and have a positive effect on patient care outcomes.

However, despite the positive effects of critical reflection, studies on critical reflection in nursing remain limited. Research identifying the concept of reflection [10,11], exploring the level of reflective thinking as a factor in improving nursing competency, and confirming the relationship between them [12] has been focused on the concept of comprehensive reflection, which includes critical reflection. Further, there are studies confirming the positive educational effects of critical reflection, such as helping improve critical thinking and nursing practice competencies [4,13,14]. Another study reported that critical reflection using reflective journaling as an educational strategy is effective in improving critical thinking disposition and problem-solving abilities [15]. Most nursing studies primarily measured self-reflection, which resulted in the improvement of clinical competence in nursing students [16]. Furthermore, a correlation between clinical reasoning competency was confirmed [17]. In previous studies, the level of critical reflection was evaluated and analyzed based on qualitative data collected through reflection journals and interviews [18,19]. Rather than reflecting the clinical situation of nurses through critical reflection, the scales used measured general self-reflection or reflective thinking, such as self-reflection and insight [20], and reflective thinking [21]. These scales were developed and widely used in the fields of pedagogy, psychology, and business administration [20,21,22,23]. It may be generally suitable for measuring overall reflection, but it is limited in measuring critical reflection competency that reflect the specific situations of clinical nursing practice in clinical settings. Although critical reflection is widely recognized as a crucial element in individual and organizational learning in nursing education, not many instruments exist to measure critical reflection in the context of nursing care.

Critical reflection in nursing is a process that connects theory or research with practice by transforming thoughts from an existing situation to a new one and converting intuition into knowledge through an in-depth understanding of the situation [24]. Critical reflection is the highest level of reflection that includes the aspect of recognizing problem situations and finding solutions through critical thinking [25]; it is different from reflection centered on individual behavior and consciousness. However, despite these differences, many nursing studies tend to use critical reflection and reflection without distinction [26]. In addition, most reflection-related scales were mainly used for self-reflection and insight to evaluate one’s own thoughts, emotions, and actions, rather than for critical reflection [20,27]. As such, they were limited to a comprehensive evaluation of critical reflection. Since critical reflection can promote critical thinking and clinical reasoning competency, which are essential for nurses’ role performance, it is crucial to measure the critical reflection of clinical nurses. Up to now there has been a lack of scales to identify differences in individual capabilities of critical reflection in clinical nursing care situations. Therefore, it was necessary to develop more sensitive and specific scales to measure critical reflection competency that reflects the clinical situation and context of the specific field of nursing. In this study, we aimed to develop a scale for measuring the critical reflection competency of clinical nurses and to verify its validity and reliability.

## 2. Materials and Methods

### 2.1. Study Design

This was a methodological study to develop and validate a scale to measure the critical reflection competency of clinical nurses.

### 2.2. Study Methods

#### 2.2.1. Scale Development

1. Concept Definition and Preliminary Item Composition: The concept of critical reflection and the components of the scale were identified through a literature review related to critical reflection and the results of previous research [3] of the content analysis on critical reflection by this research team. The results of the literature review and previous research showed that critical reflection competency is a process of restructuring through connection with prior experiences by contemplating the meaning of experiences in clinical situations. It was found that the factor that promotes critical reflection is the improvement of confidence through open mindedness and positive feedback. Based on the concepts and characteristics derived in this way, a total of 64 preliminary items were constructed to measure critical reflection competency.
2. Content Validity: The adequacy of vocabulary and expressions for the 64 preliminary items was checked with the advice of an expert from the National Institute of Korean Language. Content validity was conducted on the 64 items by an eight-member panel of experts, comprising of two nursing professors, and six clinical nurses with experience in critical reflection training. Its validity was evaluated using the content validity index (CVI) according to the criteria that a CVI of 0.78 or higher is appropriate in the case of 6 to 10 experts [28].
3. Selection of a Response Format: The Likert scale was used as a response scale for the preliminary items. A scale with less than four categories is too small, and one with more than six is difficult to distinguish [29]. Therefore, in this study, a five-point Likert scale was used, ranging from one point (“not at all”) to five points (“strongly agree”).

#### 2.2.2. Scale Validation

In this phase, the validity and reliability of the preliminary scale were verified to confirm the critical reflection competency scale for clinical nurses.

1. Sample: The participants of this study were clinical nurses with more than one year of experience working in a medical institution, and the sample was extracted by the convenience sampling method. If the sample size required for factor analysis for construct validity verification is 200 or more, it is evaluated as stable [30]. Based on the evidence that a sample size 3 to 20 times the number of items is appropriate [31], 301 participants were required for the study, considering the dropout rate of 15% based on 256, which is a quadruple of 64 questions. A total of 298 participants responded to the survey, of which the data of 296 were used for the final analysis, as one respondent did not want to participate in the study, and another had less than one year of clinical experience.
2. Measures: A preliminary scale derived through this study, a scale for critical thinking disposition [32], and a scale for clinical reasoning competence [33] were used. Critical thinking disposition is the motivation, desire, or attitude to think critically and value critical thinking [32]. The scale for critical thinking disposition is intended to measure the affective domain of critical thinking. Each subfactor had five questions on “intellectual eagerness/curiosity”, four questions on “prudence”, four items on “self-confidence”, three items on “systematicity”, four items on “intellectual fairness”, four items on “healthy skepticism”, and three items on “objectivity”, consisting of a total of 27 items, seven factors, and a five-point Likert scale. At the time of development, the Cronbach’s α was 0.84 [32], and it was 0.77 in this study. For the Korean version of the nurse clinical reasoning competence scale, the Nurse Clinical Reasoning Competence (NCRC) scale, which was developed by Liou et al. [34] and translated into Korean by Joung and Han [33], was used to verify validity and reliability. It consisted of a total of 15 items of one factor on a five-point Likert scale. The Cronbach’s α was 0.93 in the study of Joung and Han [33] and 0.81 in this study.
3. Data Collection: An online survey was conducted from 13 September to 1 November 2021. The purpose and method of the study were explained to the nursing department of the institution that provided critical reflection education, and the recruitment document for participants was posted after permission was obtained to collect data. In addition, data were collected by posting recruitment documents on the online community for nurses. Recruitment documents included the purpose and method of the study, the period and procedure for participation, compensation for loss of hours during participation, and the URL of the online questionnaire. The duration of the survey was between 10 and 15 min.
4. Data Analysis: Data were analyzed using SPSS/WIN Statistics 27.0, and frequency and percentage, mean and standard deviation were calculated by performing frequency analysis and descriptive statistics for the general characteristics of participants. Item analysis and exploratory factor analysis were performed to verify the construct validity of the scale. For item analysis, the corrected item-to-total correlation coefficient and the change in Cronbach’s α value when an item was deleted were analyzed. A correlation coefficient of less than 0.40 meant that the item had a low degree of discrimination [35]. Further, the sample fit of Kaiser-Meyer-Oklin (KMO) was checked and Bartlett’s sphericity test performed [36] to determine its suitability for factor analysis. As a factor estimation method, an exploratory factor analysis was performed using the principal component analysis method by varimax rotation, which is an orthogonal rotation. According to Kaiser’s rule, the eigenvalue of the sample correlation matrix was set to be 1.0 or more, and the criterion for each factor was 0.40 or more factor loading and 0.30 or more in communality [37]. Furthermore, for the criterion-related validity test of the scale, the correlation between the scale and critical thinking disposition scale and the scale and clinical reasoning competence scale were analyzed using the Pearson correlation coefficient. Critical thinking is an essential element for clinical reasoning, and the two scales can be seen as measuring attributes similar to the critical reflection competency. When the correlation coefficient between tools is calculated as r = 0.40–0.80, it can be considered that the criterion validity of the tool is secured [38]. The testing reliability of the scale and sub-factor scale was confirmed using Cronbach’s α for internal consistency reliability. In addition, reliability was calculated when items were removed, and the extent to which each item represents the concept to be measured was analyzed.

### 2.3. Ethical Considerations

For the ethical protection of the participants, the study was conducted after securing approval from the Institutional Review Board (IRB No. ****-202011-0005-03) of the university to which the researcher belongs. The purpose and methods of the study were explained to the participants, and a guarantee of anonymity was posted on the online survey. Written informed consent was exempted by the IRB, as participation in the survey itself was considered as consent. In addition, participants were informed that they could opt out of the survey at any time, and that data withdrawn in the middle of the survey would not be used. After the survey was completed, a mobile gift voucher was provided to the participants who agreed to the collection of their mobile phone numbers. It was explained that the archived files and completed questionnaires would be kept for three years after the end of the study, after which the documents would be discarded, and files permanently deleted in a way that they could not be restored.

## 3. Results

### 3.1. General Characteristics

The general characteristics of the participants are shown in Table 1. Of them, 95.9% were women, with the average age being 33.13 years. A total of 37.5% had more than 5 years and less than 10 years of clinical experience, with an average experience of 94.86 months. In addition, 28.7% had experienced critical reflection education, and 81.2% of such participants had used critical reflection in clinical practice.

### 3.2. Validity

#### 3.2.1. Content Validity

The item-level content validity index (I-CVI) of the preliminary items was 0.88–1.00, and none of the questions showed an I-CVI of less than 0.78. S-CVI/Ave (averaging), the average of the scale-level content validity index (S-CVI) was 0.96, which was above the standard value of 0.90. Accordingly, 64 preliminary items were derived without correction or deletion.

#### 3.2.2. Construct Validity

**Figure 1:**
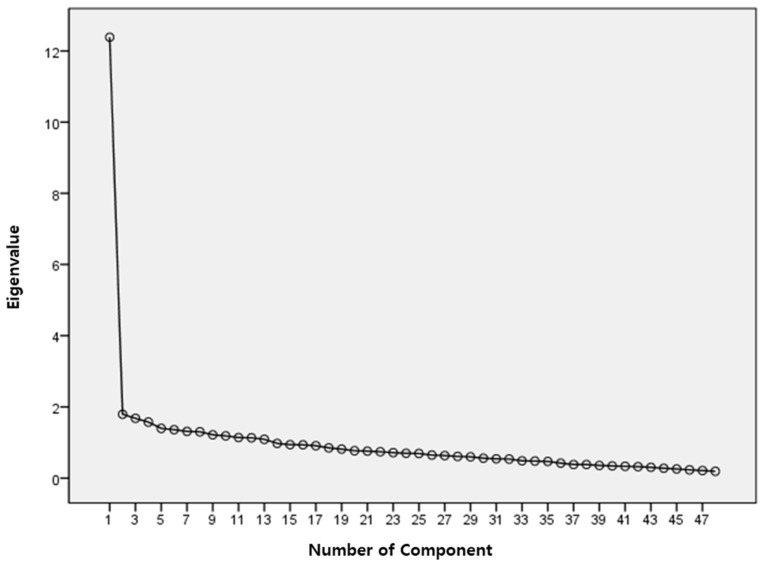
Scree Plot Eigenvalues of Exploratory Factor Analysis.

1. Exploratory Factor Analysis: Based on the item analysis, the corrected item-to-total correlation coefficient was r = 0.292–0.650. A total of 16 items with a correlation coefficient of less than 0.40 between the items and the total score were deleted, and an exploratory factor analysis was performed on 48 items. KMO and Bartlett’s sphericity test were performed to determine its suitability for factor analysis. In this study, the KMO value was 0.888, which was higher than the standard 0.50 [36]. As per the result of Bartlett’s sphericity test = 5002.958, df = 1128 (<0.001), the null hypothesis was rejected, confirming that the data were suitable for factor analysis. As a result of the first exploratory factor analysis using the principal component analysis method by varimax rotation, the number of factors with eigenvalues greater than or equal to 1.0 was found to be 13. However, as a result of referring to the Scree plot, the slope was found to have changed gently based on factor five. As such, it was judged that five factors would be derived, and the analysis was carried out by fixing the number of factors to five (Figure 1).

Ten items showed a factor loading of 0.40 or less in all factors, and three items showed a communality of 0.30 or less. There were three items with two or less items per factor, and 11 items were deleted according to the judgment of the research team for the cross-loading items, and a total of 29 items were deleted through an iterative factor analysis process. Finally, five factors were extracted from the final 19 questions and were found to explain 53.02% of the total variance (Table 2).

2. Criterion-related Validity: A correlation analysis was conducted between the scores of the critical thinking disposition scale, the clinical reasoning competence scale, and the scale developed in this study. The result of the criterion-related validity analysis showed that the correlation coefficient between the total score of the scale and the total score of the critical reflection competency scale was 0.726, indicating a significant correlation, and the criterion-related validity of the scale was secured (Table 3). The correlation between each sub-factor was found to have a significant correlation, except for the “prudence” factor. It showed a correlation of 0.40 or higher with the factors of “intellectual eagerness/curiosity,” “intellectual fairness,” and “objectivity.” The correlation coefficient between the total score of the scale and the clinical reasoning competence scale’s total score was also significant at 0.774, thus securing the basis for the criterion-related validity of the scale (Table 3). The correlation coefficients between the sub-factors were all above 0.40.

### 3.3. Reliability

The reliability, measured by Cronbach’s alpha, was 0.853 for the whole scale, with the different factors varying from 0.515–0.738 (Table 4).

## 4. Discussion

This study aimed to develop a scale to measure the critical reflection competency of clinical nurses and to verify its validity and reliability. The study developed a total of 19 items with a Cronbach’s α of 0.853, ensuring internal consistency. To verify the criterion-related validity, the correlation between critical thinking disposition and clinical reasoning competence was analyzed; the results were statistically significant at 0.726 and 0.774, respectively. According to Cohen’s criteria [39], a high correlation was established, thus confirming it as a valid scale for measuring the critical reflection competency of clinical nurses. This is consistent with the results of previous studies that reflection positively effects the improvement of critical thinking disposition [40,41,42]. However, the factor for which a statistically significant correlation was not confirmed among the sub-factors of the critical thinking disposition scale was “prudence”. Based on the results of previous studies [3], critical reflection in nursing clinical education is defined as a cyclical process leading to learning that recognizes problems, reconstructs experiences and brings changes through deliberation. This is because the scale pursues changes in critical reflection and measures continuous and cyclical characteristics, rather than the aspect of prudence, which suspends judgment until sufficient evidence is secured and persistently pursues results [32].

Following factor analysis, five factors were extracted from the final 19 items in this study. However, through literature review, the concept of critical reflection was deduced as a continuous process, which involved contemplating the meaning of experiences in clinical situations and restructuring them through connection with prior experiences. In addition, as factors that promote critical reflection, the improvement of confidence through an open mind and positive feedback was identified, and items were written based on this. Therefore, this study was based on the conceptual framework that defined critical reflection as an organically combined cyclical process rather than as a step-by-step process with independent components. Furthermore, developing a single-factor scale without clearly dividing the five factors was determined to be reasonable. It is recommended that the final 19 items be used as a single-factor scale.

Among the 64 preliminary items, certain items were removed due to the low corrected item-to-total correlation coefficient in the item analysis; they included “I participate in education such as conferences and workshops to improve nursing work competency”, and “I participate in research activities for the development of nursing work performance”. These items were derived as it was deemed necessary to expand the practical problem to the research and theoretical realm. However, most nurses have little experience in conducting research [43]. There are barriers to the use of research in clinical settings, such as lack of time to participate in research or read research work, and lack of autonomy to utilize and apply the results of research [44]. As such, it is considered to be the result of reflecting the passive aspect of nurses’ participation in research for linking research with clinical practice.

In addition, among the items removed, many were related to work errors such as “I take other people’s mistakes as a cornerstone of accountability”, “I do not cover up mistakes or act defensively”, “I check whether routine nursing tasks are based on evidence”, “I propose alternatives from various perspectives on the nursing phenomenon”, and “If I have any questions during work, I do not hesitate to ask”. These items included content that raised questions about routine work, actively expressed problems, and suggested alternatives. This suggests that the cultural characteristics that emphasize standardized work and place importance on hierarchy may have influenced it. Results of previous studies suggest that among the characteristics that cause conflict in a nursing organization, the hereditary hierarchical structures that tend to force conformity also lead to giving up on solving the problem of unjust customs [45,46].

The critical reflection competency scale was developed based on a focus group interview [3] with nurses who received training on critical reflection and used it in the training of new nurses. The items of the scale were derived from the qualitative data of clinical nurses with in-depth understanding of the concept of critical reflection. As such, the properties of critical reflection in clinical situations were well reflected. Critical reflection in a nursing clinical situation can be said to be a way for nurses to look back on their own practical actions, derive the contextual meaning of the situation they experienced, and change it to a desirable practical direction [2,4]. Unlike the existing scales that focused on self-reflection [20,47], the critical reflection competency scale included items about nursing activities and reflection on work in clinical situations. These items included “I think about the reason for the importance of nursing care implemented in the line of duty based on evidence”, “I think deeply about what I find important about my work”, “I think about the nursing care that I will be providing before I actually provide it”, among others. In addition, the reflection-in-action aspect of reflecting on one’s work during nursing practice, and the reflection-on-action aspect of reflecting on the work performed after completing the nursing activities [2] was included. These items consisted of, “I implement nursing care while keeping its purpose in mind”, “I think specifically about the outcomes of nursing care”, ”I look back on the tasks I have carried out and identify things I did well and things I did badly”, “I look back on the nursing care that I provide based on my experiences”, and “I give meaning to nursing work and feel rewarded for it”. In addition, it is thought that the items reflecting that nurses learn based on their experiences in clinical situations and change to a desirable practical direction can measure critical reflection competency in an integrated way. These items were, “I make efforts to apply the work-related knowledge that I have learned to my nursing practice”, and “I apply what I have learned from experience to future work situation”.

Only 28.7% of the participants responded that they had received education related to critical reflection. It can thus be inferred that the education on critical reflection, which connects theory and practice based on experience, and has been confirmed as an effective educational means for developing the nursing profession [24,48], is not widely used. Additionally, the developed scales were for nurses with in-depth understanding of critical reflection. However, as shown in this survey, more than half the participants had no experience in critical reflection education. The limitation of this study is that it is possible that the responses were given without an in-depth understanding of critical reflection. Therefore, it is necessary for future studies to verify construct validity by conducting a confirmatory factor analysis targeting nurses who have understanding of critical reflection. Further, it is necessary to reconfirm the validity of this scale through a study that tests the difference in critical reflection competency according to the experience of critical reflection education. In addition, we propose an experimental study to provide critical reflection education and test its effectiveness. Also, most of the participants in this study were women; gender differences in critical reflection may not have been considered. Studies are needed to determine whether there are gender differences in critical reflection ability through expanding the sample size of male nurses. Lastly, since the participants of this study were nurses working in South Korea, it is necessary to validate of this scale by reflecting various cultural contexts and realities.

## 5. Conclusions

Clinical nurses need critical thinking ability to make accurate nursing decisions based on empirical evidence in clinical situations, and critical reflection competency to look at problems from a new perspective. Improvement of critical reflection competency can positively influence not only the individual growth of nurses but also the outcomes of patient care. Therefore, it is necessary to measure the nursing-specific critical reflection ability sensitively in nursing care situations. However, the existing scales for measuring reflection have limitations in reflecting the characteristics of clinical and nursing situations. Therefore, to develop a validated scale to measure critical reflection competency by reflecting the clinical situation and context in the nursing field, this study analyzed the concept of critical reflection in clinical nurses and developed a critical reflection competency scale. In addition, its validity and reliability were verified. The items of this scale extracted for measuring critical reflection ability in nursing care situations from the interview of nurses having experience of critical reflection. Therefore, it can measure critical reflection ability in a nursing situation more sensitively than other scales. It can be used to validate the effectiveness of nurses’ educational programs or nurses’ critical reflection competency in clinical settings. The results of this study can promote the use of critical reflection as an educational strategy for nurses and provide fundamental knowledge for the development of nursing educational programs that can further improve the quality of nursing care.

## Figures and Tables

**Table 1:** General Characteristics (*N* = 296).

Variable	Category	*n*	%	Median	M ± SD
Gender	Female	284	95.9		
	Male	12	4.1		
Age (years)	20–29	74	25.0		33.13 ± 4.92
	30–39	188	63.5	33	
	40≤	34	11.5		
Education	Associate	11	3.7		
	Bachelor	222	75.0		
	Master or more	63	21.3		
Clinical experience (years)	<3	37	12.5		94.86 ± 64.95 (months)
	3–5	70	23.6		
	6–9	111	37.5		
	10≤	78	26.4		
Experiences with critical reflection education	Yes	85	28.7		
	No	211	71.3		
Experiences with using cases of critical reflection in clinical practice (*n* = 85)	Yes	69	81.2		
	No	16	18.8		

**Table 2:** Items and Factor Loadings from Exploratory Factor Analysis (*N* = 296).

No	Items	Communality	Factor Loading				
			Factor 1	Factor 2	Factor 3	Factor 4	Factor 5
1	I apply what I have learned from experience to future work situations.	0.593	0.722	0.151	0.016	0.119	0.188
2	I think about the nursing care that I will be providing before I actually provide it.	0.657	0.709	0.165	0.052	0.275	0.222
3	I think about what I can do for patients in addition to my assigned tasks.	0.594	0.612	0.324	0.172	0.241	0.161
4	I respect other people’s opinions that are different from mine.	0.593	0.587	−0.062	0.484	−0.097	−0.035
5	I think deeply about what I find important about my work.	0.573	−0.038	0.734	0.093	0.085	0.128
6	I implement nursing care while keeping its purpose in mind.	0.523	0.154	0.683	0.052	0.166	0.054
7	I look at the bigger picture when dealing with patients, rather than focusing on individual tasks.	0.409	0.161	0.543	0.241	0.173	−0.007
8	I look back on the tasks I have carried out and identify things I did well and things I did badly.	0.469	0.252	0.494	0.186	−0.105	0.340
9	I ask questions about things I do not know, and endeavor to solve them myself.	0.369	0.367	0.455	0.004	0.090	0.136
10	I give meaning to nursing work and feel rewarded for it.	0.564	0.131	0.264	0.684	0.075	0.060
11	I understand my strengths and weaknesses as a nurse.	0.567	−0.076	0.106	0.667	0.273	0.174
12	When a problem occurs, I identify the cause.	0.426	0.358	0.135	0.490	0.104	0.172
13	I make efforts to apply the work-related knowledge that I have learned to my nursing practice.	0.500	0.108	0.143	0.052	0.670	0.127
14	I listen to other people’s opinions.	0.475	0.097	0.098	0.298	0.606	−0.002
15	I acknowledge the need for me to change in the interest of self-development.	0.606	0.089	0.072	−0.068	0.579	0.503
16	When a problematic situation arises, I try to identify the behavior that caused the problem.	0.514	0.403	0.136	0.174	0.543	−0.089
17	I look back on the nursing care that I provide based on my experiences.	0.625	0.107	0.269	0.209	−0.060	0.703
18	I think about the reason for the importance of nursing care implemented in the line of duty based on evidence.	0.665	0.194	−0.180	0.397	0.184	0.635
19	I think specifically about the outcomes of nursing care.	0.351	0.198	0.261	−0.046	0.160	0.465
	Eigenvalue		5.29	1.36	1.19	1.63	1.07
	Explained variance (%)		27.86	7.16	6.26	6.12	5.62
	Cumulative explained variance (%)		53.02				

**Table 3:** Correlation among critical reflection competency, critical thinking disposition, and clinical reasoning competence (*N* = 296).

		Critical Reflection Competency					
		Total	Factor 1	Factor 2	Factor 3	Factor 4	Factor 5
Critical thinking disposition	Total	0.726 **	0.539 **	0.606 **	0.511 **	0.505 **	0.531 **
	Intellectual eagerness/ curiosity	0.657 **	0.471 **	0.558 **	0.411 **	0.458 **	0.538 **
	Prudence	0.046	0.010	0.060	0.047	0.026	0.027
	Self- confidence	0.480 **	0.353 **	0.446 **	0.320 **	0.300 **	0.346 **
	Systematicity	0.330 **	0.255 **	0.270 **	0.241 **	0.245 **	0.211 **
	Intellectual fairness	0.664 **	0.537 **	0.509 **	0.523 **	0.481 **	0.411 **
	Healthy skepticism	0.416 **	0.305 **	0.356 **	0.256 **	0.283 **	0.339 **
	Objectivity	0.607 **	0.446 **	0.459 **	0.478 **	0.435 **	0.452 **
Clinical reasoning competence	Total	0.774 **	0.623 **	0.608 **	0.558 **	0.545 **	0.535 **

** *p* < 0.01.

**Table 4:** Number of Items and Reliability (*N* = 296).

Factors	No. of Items	Cronbach’s α
Factor 1	4	0.738
Factor 2	5	0.670
Factor 3	3	0.572
Factor 4	4	0.607
Factor 5	3	0.515
Total	19	0.853

## Data Availability

The data are not publicly available due to the information contained that could compromise the privacy of research participants.
